# The Impact of Participation in Online Cancer Communities on Patient Reported Outcomes: Systematic Review

**DOI:** 10.2196/cancer.7312

**Published:** 2017-09-28

**Authors:** Mies C van Eenbergen, Lonneke V van de Poll-Franse, Peter Heine, Floortje Mols

**Affiliations:** ^1^ Department of Research Netherlands Comprehensive Cancer Organisation Utrecht Netherlands; ^2^ Division of Psychosocial Research & Epidemiology The Netherlands Cancer Institute Amsterdam Netherlands; ^3^ Stichting kanker.nl Amsterdam Netherlands; ^4^ Department of Medical and Clinical Psychology Tilburg University Tilburg Netherlands

**Keywords:** cancer, survivors, patient reported outcomes, Internet, support groups

## Abstract

**Background:**

In recent years, the question of how patients’ participating in online communities affects various patient reported outcomes (PROs) has been investigated in several ways.

**Objectives:**

This study aimed to systematically review all relevant literature identified using key search terms, with regard to, first, changes in PROs for cancer patients who participate in online communities and, second, the characteristics of patients who report such effects.

**Methods:**

A computerized search of the literature via PubMed (MEDLINE), PsycINFO (5 and 4 stars), Cochrane Central Register of Controlled Trials, and ScienceDirect was performed. Last search was conducted in June 2017. Studies with the following terms were included: (cancer patient) and (support group or health communities) and (online or Internet). A total of 21 studies were included and independently assessed by 2 investigators using an 11-item quality checklist.

**Results:**

The methodological quality of the selected studies varied: 12 were of high quality, eight were of adequate quality, and only one was of low quality. Most of the respondents were women (about 80%), most with breast cancer; their mean age was 50 years. The patients who were active in online support groups were mostly younger and more highly educated than the nonusers. The investigated PROs included general well-being (ie, mood and health), anxiety, depression, quality of life, posttraumatic growth, and cancer-related concerns. Only marginal effects—that is, PRO improvements—were found; in most cases they were insignificant, and in some cases they were contradictory.

**Conclusions:**

The main shortcoming of this kind of study is the lack of methodological instruments for reliable measurements. Furthermore, some patients who participate in online communities or interact with peers via Internet do not expect to measure changes in their PROs. If cancer survivors want to meet other survivors and share information or get support, online communities can be a trustworthy and reliable platform to facilitate opportunities or possibilities to make this happen.

## Introduction

Online social networks such as Facebook and LinkedIn have become seemingly indispensable aspects of modern life. A special kind of social support is online health communities. Patients meet each other online and share information and emotions related to their illness. They can share various forms of personal information online, ranging from pure data to pure narratives, with various hybrid forms. In 1996, the Association of Cancer Online Resources (ACOR) [1] started facilitating cancer patients online by providing a platform for them to share their experiences and other information (mainly personal narratives). People write about their illness and share experiences about living with it on a day-to-day basis in a story-form; there is little to no requesting or storage of personal data. In 2004, PatientsLikeMe (PLM) [2] was established as a community in which patients can share their medical data. PLM standardizes the information to be shared, follows the course of each patient’s illness process, stores that data in a structured database, and gives direct feedback in the form of figures on the course of the patient’s illness, also in comparison with others on the platform.

Research by ACOR has shown that patients participate on such platforms primarily to share information on their illness with each other and not so much to share their emotions [3]. PLM studies have shown that patients seek others with similar disease characteristics [4]. Community members report benefits in decision making and symptom management, which may be related to their website use [5].

The concept of *online community* has developed in recent years as a result of improved technical possibilities. Relevant literature reviews cite various forms of online contact between patients, including bulletin boards, closed networks, mailing lists, newsgroups, communities, discussion forums (moderated or otherwise), chat rooms, Facebook groups, Twitter follow groups, email groups, and so on [6-9]. Furthermore, people have come to relate to such online platforms in novel ways, partly because of the popularity of Facebook (which was launched in 2004) and other social media networks.

The term *online communities* is not well defined in the literature, although there have been various attempts to describe the phenomenon, including the definition by Rheingold: “Virtual communities are social aggregations that emerge from the Net when enough people carry on those public discussions long enough, with sufficient human feeling, to form webs of personal relationships in cyberspace” [10]. For online communities, it should be noted that communication is electronic and independent of place and time and that such communities are usually open to new members, who can register for free. By participating, people gain insight into their illness and the opportunity to connect with others in comparable circumstances [3,11].

There are many online health communities with their own specific aims. As a potentially life-threatening illness, cancer raises a wide range of specific informational and emotional support issues, which is why we specially focus on cancer communities. In recent years, the effect of participating in online communities on different outcomes of interest has increasingly been investigated. However, as yet, there has been no summarizing overview of the most significant effects of participation.

This type of research can roughly be divided into two main variants: in the first, researchers ask community participants to complete one or more questionnaires, thereby measuring the effect on the individual; and in the second, researchers analyze content that has been produced by members—a process known as *content analysis.* This systematic review corresponds to the first variant and seeks to answer the following research questions:

1. Does the literature provide evidence of improvement in patient reported outcomes (PROs) for cancer patients who participate in online communities?
2. What are the characteristics of patients who report effects of participating in online communities?

## Methods

### Search Strategy and Selection Criteria

For this systematic review, we searched for publications that describe the effects of participating in online communities in terms of PROs collected from participating patients. Studies that measured effects by means of content analysis were excluded. This review focused on *asynchronous* forms of online contact, whereby participants do not need to react to one another immediately. Unlike chat sessions, they do not need to be simultaneously online. In all cases in which synchronous interaction was possible, this was always supplemental to the asynchronous form. In some cases, an online community is part of a broader service provision, so that participants can also take part in other online activities. Evaluating other forms of online contact, such as online (self-management) interventions for treatment support, is beyond the scope of this review.

PubMed (MEDLINE), PsycINFO, Cochrane Central Register of Controlled Trials, and ScienceDirect were searched (last search June 2017) using the following terms: (cancer patient) and (support group or health communities) and (online or Internet). PubMed added the Medical Subject Headings terms.

Studies were included according to the following criteria: (1) if the publication was an original peer-reviewed research study (eg, no systematic reviews, book chapters, dissertations, poster abstracts, editorials, and letters to the editor); (2) if it was written in English; and (3) if Web-based interaction between peers was possible. Studies were excluded if they (1) involved patient populations other than cancer survivors, (2) studied a structured Web-based health intervention or were moderated by professionals, and (3) studied content through content analysis of the discussions.

**Figure 1:**
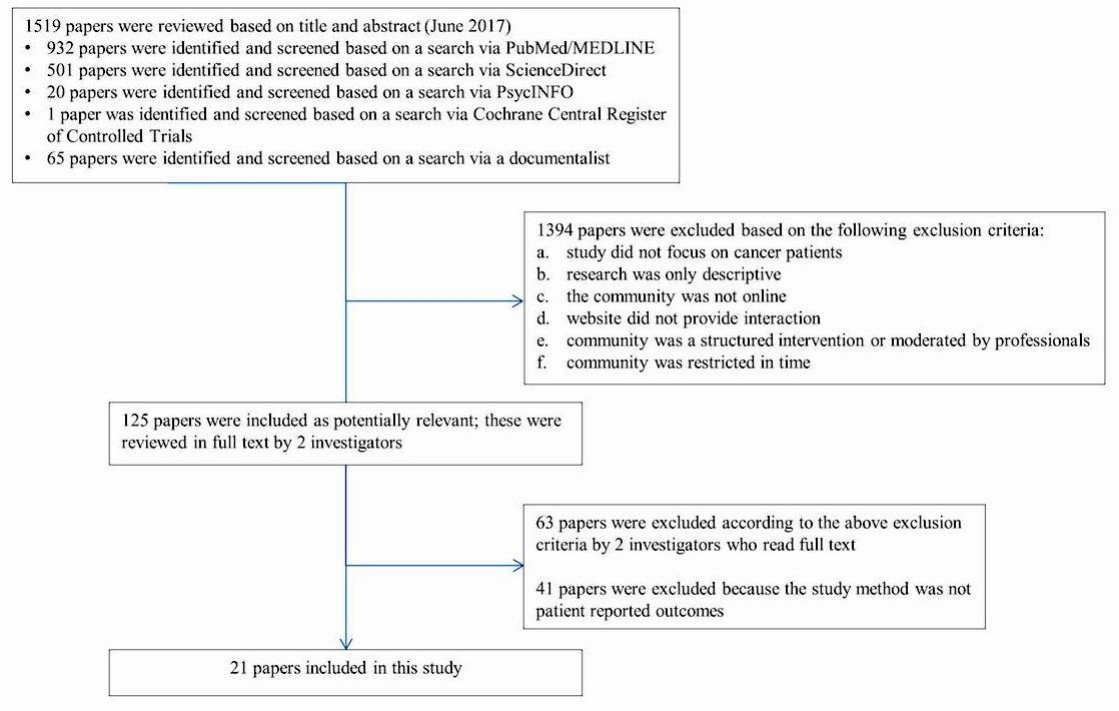
Flow chart of the literature search.

These inclusion and exclusion criteria were applied to our initial 1519 hits. After removal of duplicates and records not meeting the inclusion criteria, 125 records remained. Hard copies of these studies were obtained, and they were reviewed by 2 investigators (ME and FM) independently of each other. Both reviewers also used citation tracking to identify other studies potentially eligible for inclusion. This did not yield any new records. The 2 investigators agreed with each other on the final selection of studies: 21 were found to be eligible for inclusion in this review. Figure 1 is a flowchart of this selection procedure.

### Quality Assessment

Both investigators (ME and FM) assessed the methodological quality of each of the selected studies using an 11-item standardized checklist of predefined criteria, based on established criteria for systematic review, which are presented in Textbox 1 [12,13]. Each item of a selected study that matched our criteria received 1 point. If an item did not meet our criteria, or was described insufficiently or not at all, no point was assigned. The highest possible score was thus 11. The studies were then sorted into arbitrarily defined quality categories. Studies scoring 75% or more of the maximum attainable score (≥8 points) were considered to be of *high quality*. Studies scoring between 50% and 75% (6-7 points) were rated as being of *adequate quality.* Studies scoring lower than 50% (ie, <6 points) of the maximum attainable score were considered to be of *low quality*.

**List of criteria for assessing the methodological quality of studies.**

- A validated (quality of life [QoL] or patient reported outcome [PRO]) questionnaire is used.
- A description is included of at least two sociodemographic variables.
- A description is included of at least two clinical variables.
- Inclusion or exclusion criteria are described (patient population).
- Participation rates for patient groups are described and are more than 70%.
- Information is given about the degree of selection of sample (ratio respondents to nonrespondents).
- The study size consists of at least 50 participants (for active discussion).
- The data are prospectively gathered.
- The process of data collection is described (eg, interview or self-report).
- There is result comparison between two or more groups (eg, different chemotherapy treatments and differences in QoL for those with or without neuropathy symptoms) and/or results are compared with at least 2 time points (longitudinal vs posttreatment).
- Statistical proof for the main findings is reported.

## Results

### Study Characteristics

On the basis of our inclusion criteria, 21 studies remained for this review [14-34]. All those studies were published between 2005 and 2014, and the data collection described in them occurred between 2001 and 2011. Most of the studies, that is, 13 of them, were conducted in the United States [19-21,24-31,33,34]. With two Canadian studies [16,17], there were 15 in the English-language region. Only five of the studies were European: three in the Netherlands [14,15,18] and two in Denmark [22,23]. Only one study was conducted in a non-Western country, Japan [32].

The manner in which patients were asked to participate in the studies varied widely, including a notice on various websites [29], a community website [14,15], approaching participants in a training course [16], or a broader intervention [17,19-25,28,34]. Only in a few cases was there an explicit reference to the URL of the website where respondents were recruited [16,18,22,30].

The studies focused on the effects of participation on the patients’ informational satisfaction and emotional support. The study populations ranged from 27 [17] to 794 [23] respondents. In most of the studies, the respondents had a mean age of approximately 50 years. In 15 of the 21 studies, breast cancer communities were the object of study [14-16,19-21,24-28,31-34] so at least 80% of the study population was women.

As far as could be ascertained, validated questionnaires specifically designed for Web-based patient-to-patient contact were not available. Instead, researchers relied on existing questionnaires developed for care providers’ offline interventions toward patients or other customized questionnaires that were designed according to requirements. The studies used 29 different questionnaires (see Table 1). The most frequently used questionnaires were the Breast Cancer–Related Concerns [14,15,19,21,24,33], Functional Assessment of Cancer Therapy (FACT-B; quality of life measure for breast cancer)) [14,15,20,24,26,27], and Center for Epidemiologic Studies Depression Scale (CES-D; depression measure) [14,15,26,27,31]. The Hospitality Anxiety and Depression Scale (HADS; anxiety and depression measure) [17,25,32] and Mini-Mental Adjustment to Cancer Scale (MiniMac; mental adjustment to cancer) [14,22,23] were used fairly frequently. In many cases, a questionnaire was used only in a single study, including several custom-designed questionnaires.

### Methodological Quality of the Studies

Our assessment of the methodological quality of the 21 studies according to the list of quality criteria showed that the quality scores ranged from 4 to 11 points (Table 1), the mean quality score being 7.7. A total of 12 studies were found to be of high quality [15,17,19-25,28,33,34], though only one study received the maximum attainable score of 11 points [25]. Of the remaining nine studies, eight were of adequate quality [14,16,18,26,27,29,31,32] and one [30] was found to be of low quality according to our criteria. The studies had two general shortcomings: first, either participation rates for patient groups were not described or they were described but were less than 70% (criterion 5); second, information was not provided about the degree of sample selection (criterion 6).

### Reasons for and Impact of Participation in Online Communities

Patients participated mainly to share emotions [14-17,19-21,23,25-28,32-35] and to exchange information [16-18,20,22,24,25,28-30,32-34]. Sharing coping strategies played a limited role [14-17,31]. None of the studies referred to organizing practical help.

The research questions used in the studies varied strongly in terms of phrasing, which makes it difficult to compare the results. Some examples are as follows: *are people prepared to discuss sexuality online* [17]; *how does the behavior of posters compare with that of lurkers* [19]; *how does behavior change with time* [27]; *how do two patient groups or communities differ in behavior* [31]; and *what is the influence of family relations on participation in online groups* [34]. The study results often showed only minor differences between two groups, which in some cases were significant but in many cases contradicted each other.

### Used Instruments for Measuring PROs

The research questions—and therefore also the results—differed greatly. To present the effects that were found, we have placed the studies into two main categories, making similarities and differences more apparent. The common subject of the first category is the extent to which participating in online groups contributed to the personal well-being of the participants in question, whereas the common subject of the second category is the extent to which personal characteristics influenced online participation. Changes in personal well-being may be attributable to patients’ being able to share information [16-18,28,30] or emotions [21,23-27,31,32] with one another. Most of the studies found differences in well-being by comparing responses at two points in time, whereas some compared well-being between two different groups simultaneously. The investigated PROs ranged from screening for general well-being (ie, mood or health) through depression, anxiety, quality of life, and posttraumatic growth to cancer-related concerns. The effects found—that is, well-being improvements—were overall marginal, in most cases insignificant and sometimes contradictory. Posters were more positive than lurkers [17] and lurkers’ perceived functional well-being was significantly greater than that of posters [19]. Hoybye et al [22] found no significant difference between users and nonusers in overall quality of life or psychological well-being. Namkoong et al [28] found an effect of treatment expression and reception on emotional well-being. Those with high self-efficacy benefited more. Online mailing lists appear to be an important information source for cancer patients and also for support [30]. Patients reported that they still use online groups for informational or symptom-management needs [16]. We found no convincing evidence of improvement in PROs for cancer patients who participate in online communities.

**Table 1:** Characteristics publications and quality score.

First author, year, country	Cancer	Data collected	Study type	n	Age, in years, mean	Women, %	Questionnaires	Conclusions	Q score
Batenburg [14] 2014, Netherlands	Breast	2010	Observational	175	48	99	Breast Cancer–Related Concerns (BCRC), Center for Epidemiologic Studies Depression Scale Revised (CES-D), Emotional Approach Coping Scale (EACS), Functional Assessment of Cancer Therapy, Breast (FACT-B), Mini-Mental Adjustment to Cancer (Mini-MAC) Scale (MIMA)	Individual differences in coping influence the relationship between online support group participation and psychological well-being.	6
Batenburg [15] 2014, Netherlands	Breast	2011	Observational	125	48	100	BCRC, CES-D, EACS, FACT-B	No negative effect of online participation; more positive effect when patients approach their emotions less actively.	10
Bender [16] 2013, Canada	Breast	2008	Observational	73	56	100	Self-made	Online communities have the potential to fill gaps in supportive care.	7
Classen [17] 2013, Canada	Gynecological	2009	Observational	27	40	100	Female Sexual Distress Scale—revised (FSDS), Illness Intrusiveness Ratings Scale (IIRS), Hospitality Anxiety and Depression Scale (HADS), Self-made	Women find the intervention acceptable. Posters tend to be more positive than lurkers.	9
Frost [18] 2014, Netherlands	Unspecified	2013	Observational	115	52	55	Self-made	Patients share medical details more willingly online than daily life or identity information.	6
Han [21] 2011, USA	Breast	2001	Observational	177		100	BCRC	A combination of empathy expression and reception is crucial to obtaining optimal benefits.	10
Han [20] 2012, USA	Breast	2001	Observational	231	51	100	FACT-B	Patterns of engagement differed according to patients’ characteristics.	9
Han [19] 2014, USA	Breast	2005	Observational	325	51	100	BCRC, Partners in Health (PIH), Social support, Self-made	Patterns of engagement differed according to patients’ sociodemographic characteristics and psychosocial factors. Lurkers had a higher level of perceived functional well-being than posters at 3 months post baseline.	8
Hoybye [22] 2010, Denmark	Unspecified	2003	Observational	211	50-57	85-90	European Organization for Research and Treatment of Cancer Quality of Life Questionnaire (EORTC C300), MIMA, Profile of Mood States (POMS),	Patients not inclined to use Internet-based interventions are characterized by social position and employ more passive coping strategies.	8
Hoybye [23] 2010, Denmark	Unspecified	2004	Randomized clinical trial (RCT)	794	53-55	84-90	MIMA, POMS	Long-lasting psychological effects of participating in Internet-based support groups still need to be confirmed.	9
Kim [24] 2012, USA	Breast		Observational	177	51	100	BCRC, FACT-B	Supportive exchanges play positive, but different, roles in predicting psychosocial health outcomes. Emotional support giving and receiving tend to reinforce each other.	9
Lepore [25] 2014, USA	Breast	2011	RCT-Control group	184		100	IIRS, Self-made	The prosocial Internet support group (ISG) did not produce better mental health outcomes in distressed survivors relative to standard ISG.	11
Lieberman [27] 2005, USA	Breast		Observational	114	46	100	CES-D, FACT-B	Validation of bulletin boards as a source of support and help for breast cancer patients.	7
Lieberman [26] 2006, USA	Breast		Observational	52	46	100	CES-D, FACT-B, Posttraumatic Growth Inventory (PTGI)	Expressing certain negative emotions online is beneficial; expressing others is not.	7
Nam Koong [28] 2010, USA	Breast	2001	Observational	231	51	100	Self-made	Treatment information exchanges had a positive impact on emotional well-being for those with higher health self-efficacy but a negative influence for those with lower health self-efficacy.	10
Osei [29] 2013, USA	Prostate	2010	RCT-Control group	40	67	0	26-item Expanded Prostate Cancer Index Composite (EPIC-260), Program Satisfaction (PRSA), Relationship Satisfaction (RS), Satisfaction with Life Scale (SWL), 12-item Short-Form patient-reported survey of patient health (SF12), 36-item Short-Form Health Survey (SF36)	Providing support using Web-based methods is effective.	7
Rimer [30] 2005, USA	Unspecified	2004	Observational	362	>50	49	Information seeking items from the National Cancer Institute’s Health Information National Trends Study (HINTS), Self-made	Mailing lists appear to be an important resource for patients. Data suggest that they are perhaps underused by minority survivors.	4
Setoyama [32] 2011, Japan	Breast	2007	Observational	253		100	HADS	The results demonstrate that participating in online communities, even as a lurker, may be beneficial to patients’ mental health.	7
Seckin [31] 2011 USA	75% Breast, 25% other cancers		Observational	255		80	CES-D, Functional Assessment of Cancer Therapy (FACT), Medical Outcomes Study (MOS) Short-Form General Health Survey (SF20), Multidimensional Index of Life Quality (MILQ)	The Internet may be particularly beneficial to older adults who feel helpless to cope with cancer in old age.	7
Shaw [33] 2006, USA	Breast		Observational	144	44,5	100	BCRC, Emotional Well-being (EWB), Positive Affect Negative Affect Scale, (PANAS), Psychological General Well-Being Index (PGWBI)	Active users were more likely at pretest to consider themselves active participants in their health care.	10
Yoo [34] 2014, USA	Breast	2005-2007	Observational	111	50,9	100	60-item index of coping (COPE), Family Environment Scale (FES)	Family environment plays a crucial role in predicting participation and moderating the effects of use of online groups on coping strategies such as problem- and emotion-focused coping.	8

### Patient Characteristics Related to Effects

The studies on the influence of the various personal characteristics showed that coping strategies [14,15] and sociodemographic characteristics [19,20,22,28,33,34] influence how patients were active in an online group. On comparing active participants (posters/providers) with passive participants (lurkers/readers) and any nonusers, the age, race, socioeconomic status, and social embeddedness are revealed to influence online participation. Of the total number of respondents, 65% to 80% were younger than 60 years [30,32] or had a mean age ranging between 40 and 55 years [14,17,18,25,33,36]. Han et al [20] found a difference in mean age of 5 years between lurkers and posters and Hoybye et al [22] of 7 years between users and nonusers. However, 2 years later, the age differences between lurkers and posters had disappeared [19]. The result of Shaw’s Comprehensive Health Enhancement Support System (CHESS) study [33], in which respondents were given a computer and Internet access, is that for women with an Internet connection, the demographic differences in online participation became insignificant.

According to Han, patients with good social embeddedness are less inclined to post [20], whereas Hoybye et al [22] concluded that using the Internet does not appear to be a solution for those who experience little support in their daily lives. Users (posters and lurkers) were more likely to live alone [20], and lurkers seem to have a higher perceived well-being than posters. However, the findings suggest that lurkers and posters do not differ in their short-term health outcomes and that lurkers perform better than posters in certain outcomes because of their long-term engagement in online groups [19].

## Discussion

This systematic review showed that participation by cancer patients in online communities does not have a large effect in PROs. This review also indicated that most of the respondents in the reviewed studies were women (80%), as 15 out of the 21 studies were related to breast cancer communities. It was found that participants mainly want to share emotions and information and, in some cases, coping strategies as well. As the research questions and measurement instruments used in the studies varied strongly, it is difficult to compare their results.

### Study Characteristics

As far as can be ascertained, no exclusive validated questionnaires exist for measuring the effects of Web-based patient-to-patient contact. A total of 28 different validated or customized questionnaires were used. If a community is also part of a broader (online) program for patients [17,19-24,28,29,33,34], it is probably even more difficult to measure the effects of participating in it.

### Methodological Quality of the Studies

The studies included in this review provide only meager description of the context of the researched communities, possibly because there are few available definitions to facilitate description of differences between communities and/or categorization of their characteristics. Not only is social interaction on Internet a relatively new domain, but it is also continuously developing. In a relatively short time span (10-15 years), there have been great changes, partly because of technological developments. A community’s launch year and its available starting and running budgets largely determine the technological possibilities of the platform. As the application is almost never commercial, there is a limited budget for further development. ACOR is a prime example of this. Although it was once a pioneer, its impact has diminished in recent years because of technological limitations. The publications on this platform are from before 2010 [3,37].

This review reveals that researchers have not yet succeeded in developing a research method to assess the impact of participating in online cancer communities that, when repeated, produces results that can be compared. As yet, there is insufficient methodological framework to speak of a research field. Researchers do not even have or use a standard, agreed definition of an online community. They do not describe the characteristics of the researched communities and how these influence the research results. Presumably, the various possibilities of the technology, the graphic design, the marketing, the online and offline references to the community, the provider’s reliability, and so on, all have an impact on the user experience and may partly determine participants’ success and satisfaction, thereby influencing the research results. The impact of these factors should be measurable; otherwise it will be impossible to determine the effects of patients’ participation in Internet communities. Research into patients’ Internet use has clearly shown that personal and illness characteristics influence use [22,38]. However, it has yet to be clarified how patients’ Internet skills and expectations regarding interactive possibilities influence their experienced degree of satisfaction with the platforms and affect their psychosocial well-being. In the reviewed studies, most of the research populations were too small to take population variation into account. Zhang’s framework for organizing research of online health communities shows us how many variables can be studied [7]. Leimeister et al [39] designed a model for measuring social support in online communities, which makes it possible to compare the effects of participating in different communities for different patients.

None of the reviewed studies included an attempt to describe the software-based interactive possibilities and their influence on the results. The combination of rapid technological developments and different budgets has led to great differences between the online platforms, making comparison of results meaningless—if not impossible.

### Reasons and Impact of Participation in Online Communities

*Talking about* the illness with others who are well acquainted or less well acquainted, on the Internet or otherwise, can contribute to (learning to) deal with the reality of being seriously ill [15,40,41]. In this context, online communities can have a function, in that people are able to meet each other virtually and share experiences. However, it is difficult to objectively and quantitatively measure the effect on personal well-being by means of PROs [16-18,21,23-28,30-32]. The most commonly cited factors that influence the extent to which patients are active on Internet are demographics, including age, gender, education level, and stage of illness. In the literature, no negative effects of patients’ participating in online platforms are cited, although in some cases incorrect information has not been corrected fast enough in such environments [42]. Do online and offline forms of social contact between patients have the same advantages and disadvantages? The most important criterion of how social contact occurs should be patients’ preferences, precisely because personal characteristics influence the effects of participation in online communities [21,23-27,31,32].

### Patient Characteristics Related to Effects

It seems that the Internet has become one of the main social environments in which individuals act—to a greater or lesser degree. Whether people actually make use of the Internet is strongly determined by personal and illness characteristics, social background, needs, and various computer and Internet skills [8]. However, these variables were insufficiently taken into account in the different studies, even though they generally influence individuals’ quality of life. Although participating in an Internet community does not appear to make a big difference in improving PROs, it can add considerable value for some patients, in that they are able to connect and converse with fellow patients at any time. If patients have major concerns, the effect of participation can reasonably be expected to be greater.

The limited diversity of respondents in the studies—in particular, the large numbers of women with breast cancer—makes it difficult to treat the results as generally applicable. Figures from the Netherlands Cancer Registry [43] indicate that only about one-third of all women with cancer in 2014 had breast cancer, whereas in the reviewed studies, approximately 90% of the women had that type of cancer. Most of the respondents in the reviewed studies had a mean age of approximately 50 years, whereas in the Netherlands, for example, generally at least 70% of cancer patients are 60 years or older when first diagnosed, and, in the case of breast cancer, 80% of the patients are 50 years or older. Therefore, it can reasonably be concluded that the age distribution of the surveyed population differs from that of the general population of cancer patients and that a younger population of patients is active on the Internet.

A tentative conclusion can be drawn regarding added value for women with breast cancer, although the respondents indicated very few illness characteristics to make reliable statements regarding the total breast cancer population.

### Conclusions

Given the large number of influencing factors, in combination with the difficulty of comparison and the limited results, we conclude that there is little to be gained from further research in how participation in online community influences PROs. The conditions under which effects are obtained are difficult to reproduce. A specific model, such as described and tested by Leimeister et al [39], may be a more reliable tool for measuring the effects of participation in online communities.

Despite our conclusion, we believe that online communities are relevant for some patients who wish to communicate with their peers by writing and reading [44,45] because they think it will help them to cope with their situation. It is not unlike a *real* conversation with friends or relatives or reading a book describing a patient’s journey. Patients can interact with peers in online patient communities, exactly at their preferred time, place, and pace. The evidence for negative implications is small [44,45].

To further this development, we believe that research on standardization of infrastructure for care communities, which has proven to be workable in practice, may be appropriate at this juncture. That would enable upscaling, also for other illness patterns and in other language regions. This may be a useful and interesting concept for a major socially responsible cooperative project involving Facebook, Google, and patient organizations. Facebook has a great deal of know-how when it comes to building social networks, and Google can readily search the content; patients can test that environment for functionality and interaction.

## Abbreviations

ACOR: Association of Cancer Online Resources
PLM: PatientsLikeMe
PROs: patient reported outcomes
